# Maternal and Fetal Outcomes in Pregnant Women with Burn Injuries: A Systematic Review and Meta-Analysis

**DOI:** 10.1093/jbcr/irag155

**Published:** 2026-08-28

**Authors:** Juan Jose Parra Molina, Sergio Romero, Edgar Fabian Manrique Hernandez

**Affiliations:** Universidad Industrial de Santander, Bucaramanga, Santander, Colombia; Universidad Industrial de Santander, Bucaramanga, Santander, Colombia; Universidad Industrial de Santander, Bucaramanga, Santander, Colombia; Hospital Internacional de Colombia HIC - Fundación Cardiovascular de Colombia FCV - Fundación Universitaria FCV, Piedecuesta, Colombia

**Keywords:** burn injuries, pregnancy, maternal mortality, Fetal mortality, systematic review, meta-analysis, Total body surface area, obstetric outcomes

## Abstract

Burn injuries during pregnancy are associated with significant maternal and fetal morbidity and mortality; however, available evidence is fragmented and largely based on small observational studies.

To estimate pooled maternal and fetal mortality among pregnant women with burn injuries and explore the impact of burn severity.

A systematic review and meta-analysis of proportions was conducted following PRISMA 2020 guidelines. PubMed/MEDLINE, Scopus, Web of Science, and LILACS were searched from inception to March 2026. Observational studies and case series reporting maternal and/or fetal outcomes were included. Risk of bias was assessed using Newcastle–Ottawa Scale and Joanna Briggs Institute tools, and certainty of evidence using Grading of Recommendations Assessment, Development and Evaluation. Random-effects models with Freeman–Tukey double arcsine transformation were applied.

Results: Twenty-two studies including 1162 pregnant women were analyzed. Most studies were case series (*n* = 15), followed by cohort studies (*n* = 5) one cross-sectional study, and one case report. Maternal mortality ranged from 0% to 70%. The pooled maternal mortality proportion was 0.43 (95% CI, 0.36-0.50), with substantial heterogeneity (*I*^2^ = 72.9%). Fetal mortality ranged from 31.3% to 90.5%, with a pooled estimate of 0.57 (95% CI, 0.49-0.65; *I*^2^ = 78.6%). Burn severity, particularly TBSA burned ≥50%, was consistently associated with worse outcomes. Funnel plots suggested possible publication bias. The overall certainty of evidence was low. Burn injuries during pregnancy are associated with high maternal and fetal mortality, strongly influenced by burn severity. Despite consistent findings, the low certainty of evidence highlights the need for high-quality prospective studies.

## INTRODUCTION

Burn injuries remain an important cause of preventable morbidity and mortality worldwide, particularly in low- and middle-income countries where exposure to open flames, gas explosions, and unsafe domestic practices is common.^1–5^ Although burns during pregnancy are uncommon, they represent a high-risk condition because they affect both the mother and the fetus. These injuries are more frequently reported in resource-limited settings and are often associated with domestic accidents, self-inflicted injuries, and social vulnerability.^1–5^ The physiological adaptations of pregnancy may increase vulnerability to severe burn injury. Burn-induced hypovolemia, respiratory compromise, systemic inflammation, and infection can impair uteroplacental perfusion, resulting in fetal hypoxia, miscarriage, intrauterine fetal death, or preterm delivery.^6–9^

TBSA burned is the most important predictor of maternal and fetal outcomes. Studies consistently show increasing mortality when burn size exceeds 40%-50% TBSA, likely due to greater fluid loss, shock, infection, and multiorgan dysfunction.^10–14^ Fetal mortality often exceeds maternal mortality because of the fetus’s limited ability to tolerate maternal hypoxemia, hypotension, and sepsis. Prognosis is also influenced by gestational age, inhalation injury, sepsis, and intentional burns, which are frequently associated with delayed presentation and adverse social determinants.^15–17^

Despite its clinical importance, the evidence remains fragmented and is largely derived from retrospective studies and small cohorts, resulting in substantial variability in reported outcomes. Therefore, a systematic review and meta-analysis is needed to provide more robust estimates of maternal and fetal mortality and to clarify the relationship between burn severity and mortality outcomes. Accordingly, this study aimed to estimate the pooled proportions of maternal and fetal mortality among pregnant women with burn injuries and to evaluate the association between %TBSA burn and mortality.

## METHODS

This systematic review and meta-analysis of proportions was conducted in accordance with the Preferred Reporting Items for Systematic Reviews and Meta-Analyses (PRISMA 2020 statement). A protocol was developed a priori to define the study objectives, eligibility criteria, and analytical approach; registration was completed in PROSPERO (CRD420261384572).

We included observational studies (cohort, case–control, and cross-sectional studies) and case series reporting maternal and/or fetal outcomes in pregnant women with burn injuries. Given the expected rarity of this condition and the limited availability of high-level evidence, case series were considered eligible to maximize data capture. Studies were excluded if they involved non-pregnant populations, animal models, non-original data (reviews, editorials), or lacked sufficient information to extract outcomes of interest.

The target population comprised pregnant women with burn injuries at any gestational age and regardless of burn severity or etiology. The primary outcomes were maternal mortality and fetal mortality. Secondary outcomes included maternal complications (sepsis, multi-organ failure, intensive care unit admission), obstetric outcomes (preterm delivery), and neonatal outcomes (low birth weight, Apgar score, and neonatal morbidity). Due to anticipated heterogeneity in outcome definitions, all variables were extracted and analyzed according to the definitions provided in each study.

A comprehensive and sensitive search strategy was designed and implemented in PubMed/MEDLINE, Scopus, Web of Science, and LILACS electronic databases from inception to March 23, 2026. The search combined controlled vocabulary and free-text terms related to pregnancy and burn injuries, using Boolean operators and database-specific adaptations. No language or publication date restrictions were applied. In addition, backward citation tracking of included studies and relevant reviews was performed to identify additional eligible reports (supplement 1).

All records were imported into Rayyan, where duplicates were identified and removed.^17^ Study selection was conducted by 2 authors: initial screening of titles and abstracts followed by full-text assessment. Two reviewers independently performed each screening stage, and disagreements were resolved through discussion and consensus. No formal inter-rater agreement statistics were calculated, consistent with the exploratory nature of the screening process.

Data extraction was independently conducted by 2 reviewers using a standardized and pilot-tested data collection form in Microsoft Excel. Extracted data included study design, country, sample size, maternal age, gestational age, burn characteristics (including total body surface area and burn severity when available), and all reported maternal and fetal outcomes. When multiple reports from the same population were identified, the most comprehensive dataset was retained. Missing data were not imputed and were reported as not available.

Risk of bias assessment was performed using design-specific validated tools appropriate for observational studies. Cohort studies were evaluated using the Newcastle–Ottawa Scale,^18^ while cross-sectional studies and case series were assessed using the Joanna Briggs Institute (JBI) critical appraisal checklists.^19^ Each study was evaluated independently by 2 reviewers across relevant methodological domains, and disagreements were resolved by consensus. The overall certainty of the evidence for each outcome was assessed using the Grading of Recommendations Assessment, Development and Evaluation (GRADE) approach, considering domains of risk of bias, inconsistency, indirectness, imprecision, and publication bias.^20^

For quantitative synthesis, a meta-analysis of proportions was conducted to estimate pooled prevalence for each outcome. To address variance instability associated with proportions close to 0 or 1, the Freeman–Tukey double arcsine transformation was applied prior to pooling. Summary estimates were calculated using a random-effects model based on the DerSimonian–Laird method, given the expected clinical and methodological heterogeneity across studies. Statistical heterogeneity was assessed using Cochran’s Q test and quantified using the *I*^2^ statistic, with thresholds interpreted according to conventional criteria.

All analyses were performed using R.^21^ Where data permitted, subgroup analyses were planned according to burn severity, TBSA, and gestational age at the time of injury. Sensitivity analyses were conducted by excluding studies at high risk of bias to evaluate the robustness of pooled estimates.

Publication bias was assessed through visual inspection of funnel plots and Egger’s regression test when a minimum of 10 studies were available for a given outcome, acknowledging the limitations of these methods in meta-analyses of proportions.

## RESULTS

A total of 142 records were identified through database searching, with no additional records identified from other sources. After removal of 63 duplicates, 79 records remained for screening. Following title and abstract screening, 50 records were excluded for not meeting the predefined inclusion criteria. A total of 29 full-text articles were assessed for eligibility. During the full-text review, 7 articles were excluded, and 22 met the eligibility criteria. In total, 22 studies were included in the systematic review and meta-analysis. The study selection process is detailed in Figure 1.

**Figure 1:**
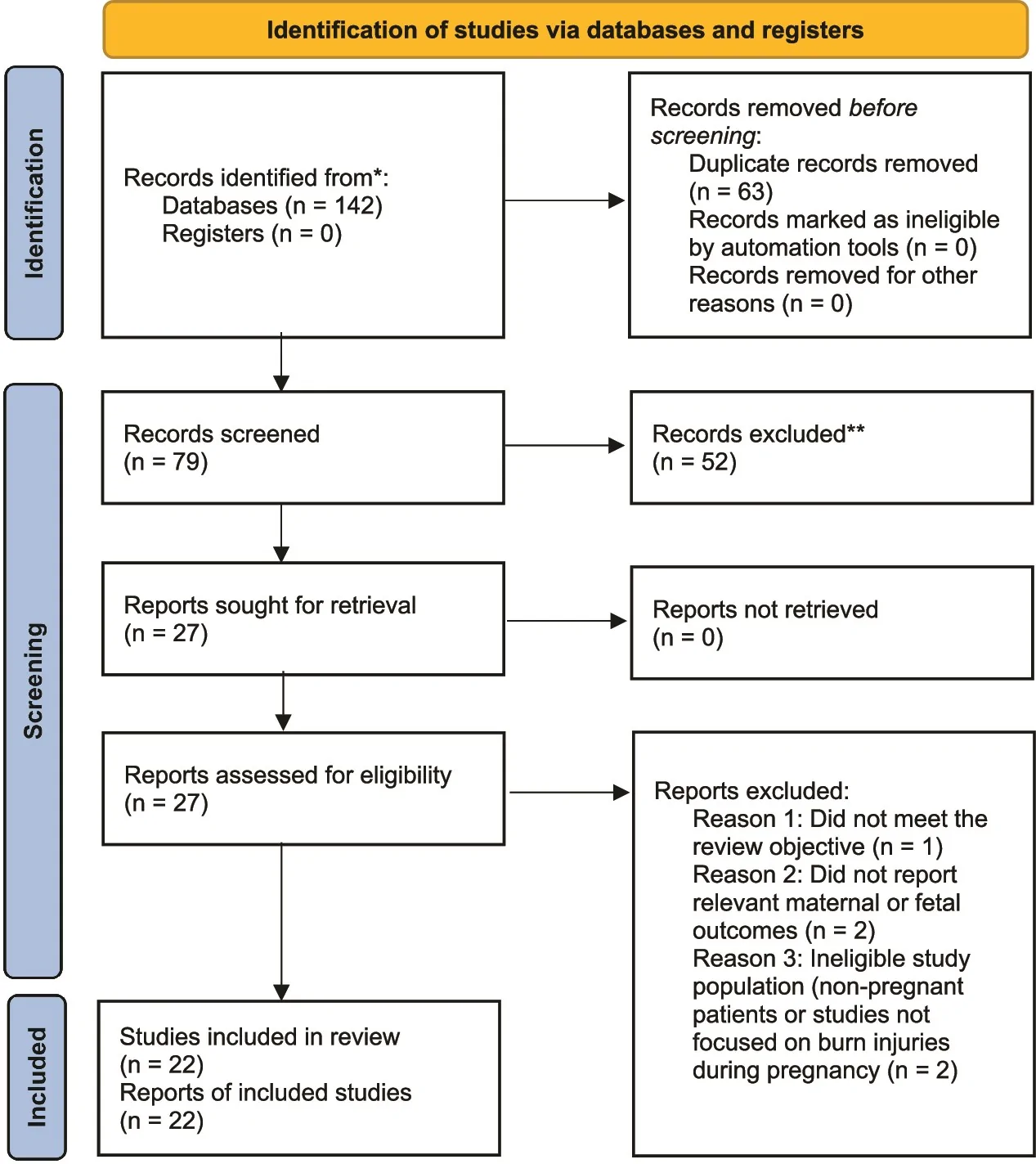
PRISMA 2020 Flow Diagram Illustrating the Study Selection Process

A total of 22 studies evaluating burns during pregnancy were included, comprising 1162 pregnant women with burn injuries (Table 1). The evidence base was predominantly composed of case series (*n* = 15), followed by cohort studies (*n* = 5), 1 cross-sectional study, and a single case report, reflecting the limited availability of higher-level analytical designs in this field.

**Table 1:** Characteristics of Included Studies on Burns During Pregnancy

Author (year)	Country	Design	*N* (pregnant)	Maternal age	Gestational age	Trimester	Burn type	TBSA burned (%)	Maternal mortality	Fetal mortality	Key findings
Ambedkar^5^	India	Cohort	22	25.2 ± 0.23	NR	Mixed	Thermal	<30->70	63.6%	68.2%	Severe burns associated with poor outcomes
Mittal^23^	India	Cohort	67	24.6	NR	Mixed	Thermal	15->70	57.8%	68.8%	High prematurity and sepsis
Pasalar^25^	Iran	Cohort	39	23.7 ± 0.48	NR	Mixed	Thermal	1-100	30.8%	38.5%	Lower mortality vs other cohorts
Akhtar et al.^10^	India	Cohort	50	NR	NR	Mixed	Mixed	10->60	70.0%	72.0%	High mortality in TBSA burned >60%
Taylor et al.^7^	USA	Cohort	19	16-37	NR	Mixed	Thermal	<10->40	36.8%	36.8%	Early evidence of TBSA effect
Rezavand et al.^24^	Iran	Cross-sectional	39	23.5 ± 0.48	23.8 ± 0.76	NR	Mixed	<25->75	66.7%	NR	High sepsis rate
Mabrouk et al.^11^	Egypt	Case series	7	19-33	NR	NR	Mixed	12-40	0.0%	42.9%	Variable outcomes in small sample
Karimi et al.^13^	Iran	Case series	89	24.1 ± 0.56	19.2 ± 0.92	Mixed	Mixed	0-100	44.9%	47.2%	Sepsis as main complication
Sapre et al.^14^	India	Case series	15	24.6^*^	22.5^*^	Mixed	Thermal	20->80	46.7%	73.3%	High mortality with TBSA burned >60%
Seyedzadeh et al.^12^	Iran	Case series	37	32 ± 6	19.5 ± 10	NR	Thermal	<25->50	43.2%	56.8%	TBSA burned strongly associated with mortality
Vaghardoost et al.^26^	Iran	Case series	38	26.5 ± 6	NR	Mixed	Thermal	1-90	47.4%	50.0%	High rate of self-inflicted burns
Karimi et al.^2^	Iran	Case series	53	22.9 ± 0.53	NR	Mixed	Thermal	1-100	52.8%	56.6%	High complication burden
Chama and Na'Aya^4^	Nigeria	Case series	21	<15-35	NR	Mixed	Thermal	>40	47.6%	90.5%	Extremely high fetal mortality
Rezavand and Seyedzadeh^22^	Iran	Case series	91	NR	NR	Mixed	Thermal	25->50	59.5%	83.5%	Very high mortality rates
Maghsoudi et al.^1^	Iran	Case series	51	24.2	NR	Mixed	Thermal	~28-42	39.2%	45.1%	Suicide burns increase mortality
Mago et al.^3^	India	Case series	386	19-35	NR	Mixed	Thermal	10-100	47.9%	65.8%	Social determinants relevant
Gang et al.^16^	Kuwait	Case series	16	18-35	8-34	Mixed	Thermal	<30->70	6.3%	31.3%	Mortality increases with TBSA burned
Unsur et al.^9^	Turkey	Case series	11	18-36	7-34	Mixed	Thermal/scald	10->50	18.2%	36.4%	TBSA burned linked to outcomes
Amy et al.^8^	USA	Case series	30	15-37	5-36	NR	Thermal	<10->40	33.3%	43.3%	Multi-organ complications
Rayburn et al.^6^	USA	Case series	30	16-41	7-39	NR	Thermal	10-100	26.7%	40.0%	Growth restriction, prematurity
Matthews^15^	UK	Case series	50	15-39	NR	NR	NR	<50->50	28.0%	NR	Prematurity frequent
Mabrouk and el-Feky^27^	Egypt	Case report	1	28	32	3rd	Thermal	41	0.0%	0.0%	Favorable outcome with C-section

Most studies were conducted in low- and middle-income countries, particularly in South Asia, the Middle East, and Africa. Maternal age was generally within the young reproductive range (approximately 19-32 years), and burn injuries were reported across all trimesters without a consistent predominance. Thermal burns were the most frequent mechanism, mainly related to flame exposure, kerosene, and domestic gas. Several studies reported a relevant proportion of intentional injuries, including self-inflicted burns, particularly in specific sociocultural contexts.

Burn severity, commonly assessed using TBSA, showed wide variability across studies, ranging from minor (<10%) to extensive injuries (>80%). However, a consistent pattern emerged in which a substantial proportion of patients presented with moderate to severe burns (TBSA burned ≥30%), with multiple studies reporting a high frequency of critical burns (TBSA burned ≥50%). Across all designs, %TBSA burn was the most consistently reported determinant of prognosis.

Maternal mortality varied markedly across studies, ranging from 0% in small case series to as high as 70% in cohort studies. Cohort studies reported maternal mortality between 30.8% and 70.0%, while case series ranged from 0% to 59.5%, while cohort and cross-sectional estimates exceeded 60%. The cross-sectional study reported a maternal mortality of 66.7%. Across all study designs, higher mortality rates were consistently observed in patients with larger %TBSA burn involvement. The main reported causes of maternal death were sepsis, hypovolemic shock, and multiorgan failure.

Fetal mortality was consistently high and, in many studies, exceeded maternal mortality. Reported rates ranged from 0% to 90.5%, with the highest values observed in studies including patients with extensive burns (TBSA burned >40%-50%). Cohort studies reported fetal mortality between 36.8% and 72.0%, whereas case series frequently reported rates above 50%. In addition to intrauterine fetal demise, other adverse outcomes included spontaneous abortion, preterm birth, and low birth weight. Prematurity and intrauterine growth restriction were recurrent findings among surviving neonates.

A strong and consistent association between burn severity and adverse outcomes was observed across all study designs. Increasing %TBSA burn was directly correlated with higher maternal and fetal mortality, as well as with the occurrence of severe complications such as sepsis and organ failure. Some studies suggested a threshold effect, particularly at TBSA ≥50% burn size, where both maternal and fetal survival declined substantially.

The methodological quality of the included studies is summarized in Table 2. Overall, the risk of bias across the body of evidence was moderate to high, largely reflecting the predominance of observational and descriptive study designs.

**Table 2:** Risk of Bias Assessment of Included Studies

Study	Design	Tool	Score/judgment	Risk of bias
Akhtar et al.^10^	Cohort	NOS	6/9 stars	Moderate
Ambedkar et al.^5^	Cohort	NOS	7/9 stars	Moderate
Mittal et al.^23^	Cohort	NOS	7/9 stars	Moderate
Pasalar et al.^25^	Cohort	NOS	7/9 stars	Moderate
Taylor et al.^7^	Cohort	NOS	5/9 stars	Moderate–high
Rezavand et al.^24^	Cross-sectional	JBI	5/8 criteria met	Moderate
Karimi et al.^13^	Case series	JBI	8/10 criteria met	Moderate
Vaghardoost et al.^26^	Case series	JBI	7/10 criteria met	Moderate
Seyedzadeh et al.^12^	Case series	JBI	8/10 criteria met	Moderate
Rezavand and Seyedzadeh^22^	Case series	JBI	7/10 criteria met	Moderate–high
Chama and Na'Aya^4^	Case series	JBI	7/10 criteria met	Moderate–high
Gang et al.^16^	Case series	JBI	6/10 criteria met	Moderate–high
Unsur et al.^9^	Case series	JBI	6/10 criteria met	Moderate–high
Sapre et al.^14^	Case series	JBI	6/10 criteria met	Moderate–high
Karimi et al.^2^	Case series	JBI	8/10 criteria met	Moderate
Maghsoudi et al.^1^	Case series	JBI	8/10 criteria met	Moderate
Mago et al.^3^	Case series	JBI	4/10 criteria met	High
Mabrouk and el-Feky^27^	Case report	JBI	7/10 criteria met	Moderate
Mabrouk et al.^11^	Case series	JBI	7/10 criteria met	Moderate
Amy et al.^8^	Case series	JBI	7/10 criteria met	Moderate
Rayburn et al.^6^	Case series	JBI	6/10 criteria met	Moderate–high
Matthews^15^	Case series	JBI	6/10 criteria met	Moderate–high

Risk of bias was assessed using design-specific validated tools. Cohort studies were evaluated using the Newcastle–Ottawa Scale (NOS), which assesses selection, comparability, and outcome domains (maximum 9 stars). Cross-sectional studies, case series, and the case report were appraised using the Joanna Briggs Institute (JBI) critical appraisal checklists. Scores represent the number of criteria met. Overall risk of bias was categorized as low, moderate, or high based on the proportion of fulfilled items and methodological limitations identified in each study.

Among the 5 cohort studies, methodological quality was generally moderate. These studies demonstrated adequate selection of participants and outcome assessment; however, limitations were identified in the control of confounding variables, particularly burn severity and associated clinical factors, resulting in Newcastle–Ottawa Scale scores ranging from 5 to 7 stars. The single cross-sectional study also showed moderate quality, meeting 5 out of 8 JBI criteria, with the main limitation being the lack of adjustment for potential confounders. The majority of the included studies were case series, which inherently carry a higher risk of bias. Most case series were classified as having moderate methodological quality, fulfilling between 7 and 8 out of 10 JBI criteria. These studies generally reported patient characteristics and outcomes adequately. Nevertheless, several recurrent limitations were observed, including retrospective design, absence of clearly defined consecutive inclusion in some cases, and incomplete or inconsistently reported follow-up.

A subset of studies was judged to have moderate-to-high risk of bias, primarily due to small sample sizes, limited methodological transparency, and incomplete outcome reporting. One study was classified as having high risk of bias, as it fulfilled only 4 out of 10 JBI criteria, reflecting substantial limitations in study design and reporting. Overall, the most frequent sources of bias across studies included lack of control for confounding factors, variability in the reporting of burn severity, and heterogeneity in clinical management. These limitations should be considered when interpreting the pooled estimates.

Figure 2 presents the pooled proportion of maternal mortality among pregnant women with burn injuries. Individual study estimates demonstrated wide variability, ranging from 0% in small case series to 70% in cohort studies, with most estimates clustering between 30% and 60%. Larger studies contributed greater weight to the pooled estimate, although high-mortality studies with extensive burn severity (particularly TBSA burned ≥50%) consistently showed higher proportions.

**Figure 2:**
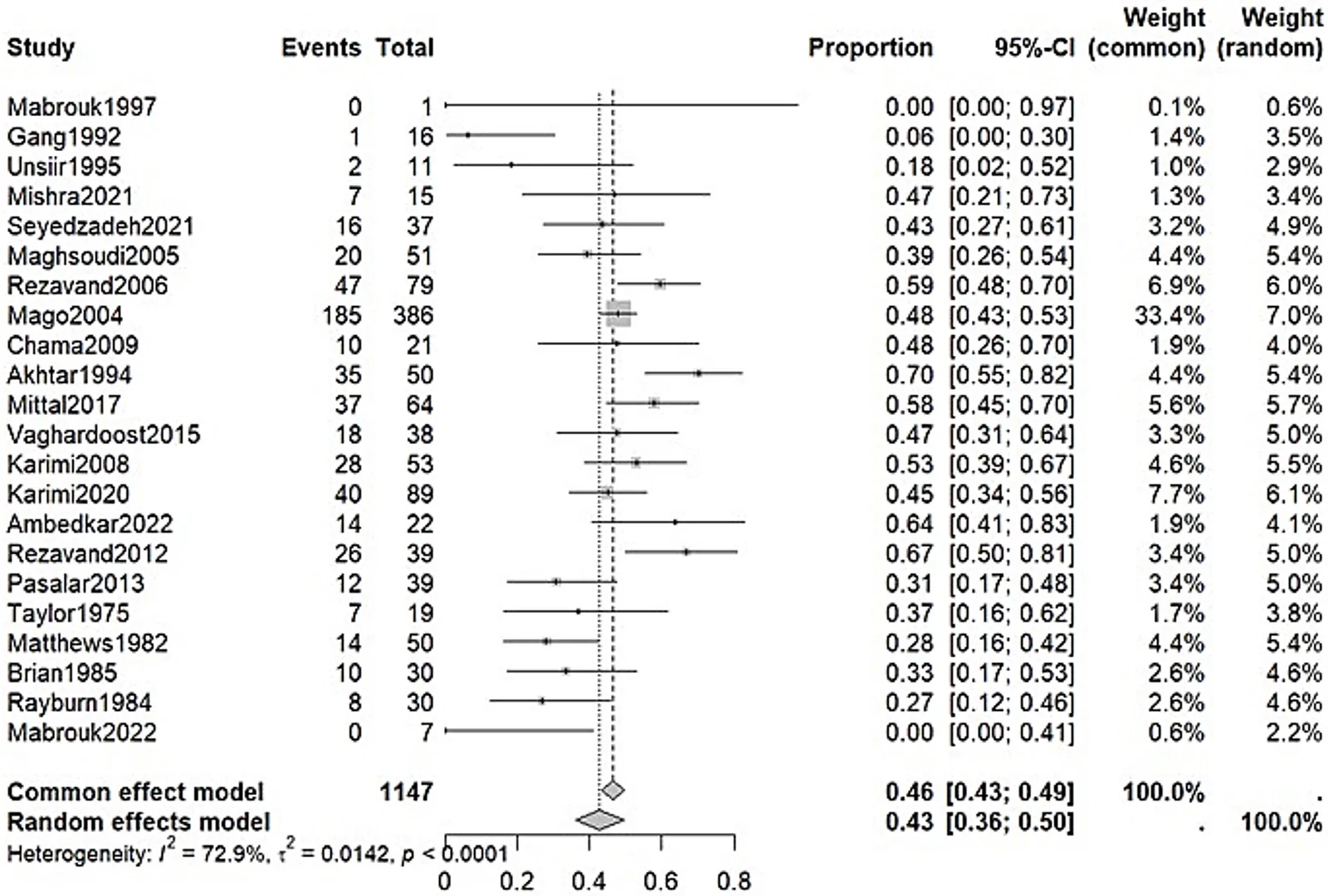
Pooled Proportion of Maternal Mortality Among Pregnant Women With Burn Injuries. Forest plot showing individual study estimates and pooled proportion of maternal mortality among pregnant women with burn injuries. A random-effects model (DerSimonian–Laird) with Freeman–Tukey double arcsine transformation was used. Horizontal lines represent 95% CIs, and square sizes are proportional to study weight. The diamond represents the pooled estimate.

Using a random-effects model (DerSimonian–Laird) with Freeman–Tukey double arcsine transformation, the pooled maternal mortality proportion was 0.43 (95% CI, 0.36-0.50). The fixed-effect model yielded a slightly higher estimate (0.46; 95% CI, 0.43-0.49), but given the presence of substantial heterogeneity, the random-effects model was considered more appropriate. Between-study heterogeneity was considerable (*I*^2^ = 72.9%, *P* < .0001), indicating significant variability across studies. This heterogeneity is likely attributable to differences in burn severity, distribution of %TBSA burn, variability in clinical management, and underlying contextual factors such as healthcare resources and the prevalence of intentional injuries. Studies including a higher proportion of patients with extensive burns consistently reported higher mortality, reinforcing the central role of burn severity as a prognostic determinant.

Overall, the pooled analysis indicates that approximately 43% of pregnant women with burn injuries die, highlighting the severe clinical burden and the need for timely, specialized management strategies in this high-risk population.

Figure 3 presents the pooled proportion of fetal mortality among pregnancies complicated by burn injuries. Individual study estimates showed substantial variability, ranging from 0% in a small case report to as high as 90% in studies including patients with extensive burn injuries. Most estimates clustered between 40% and 70%, indicating a consistently high burden of fetal loss across diverse clinical settings.

**Figure 3:**
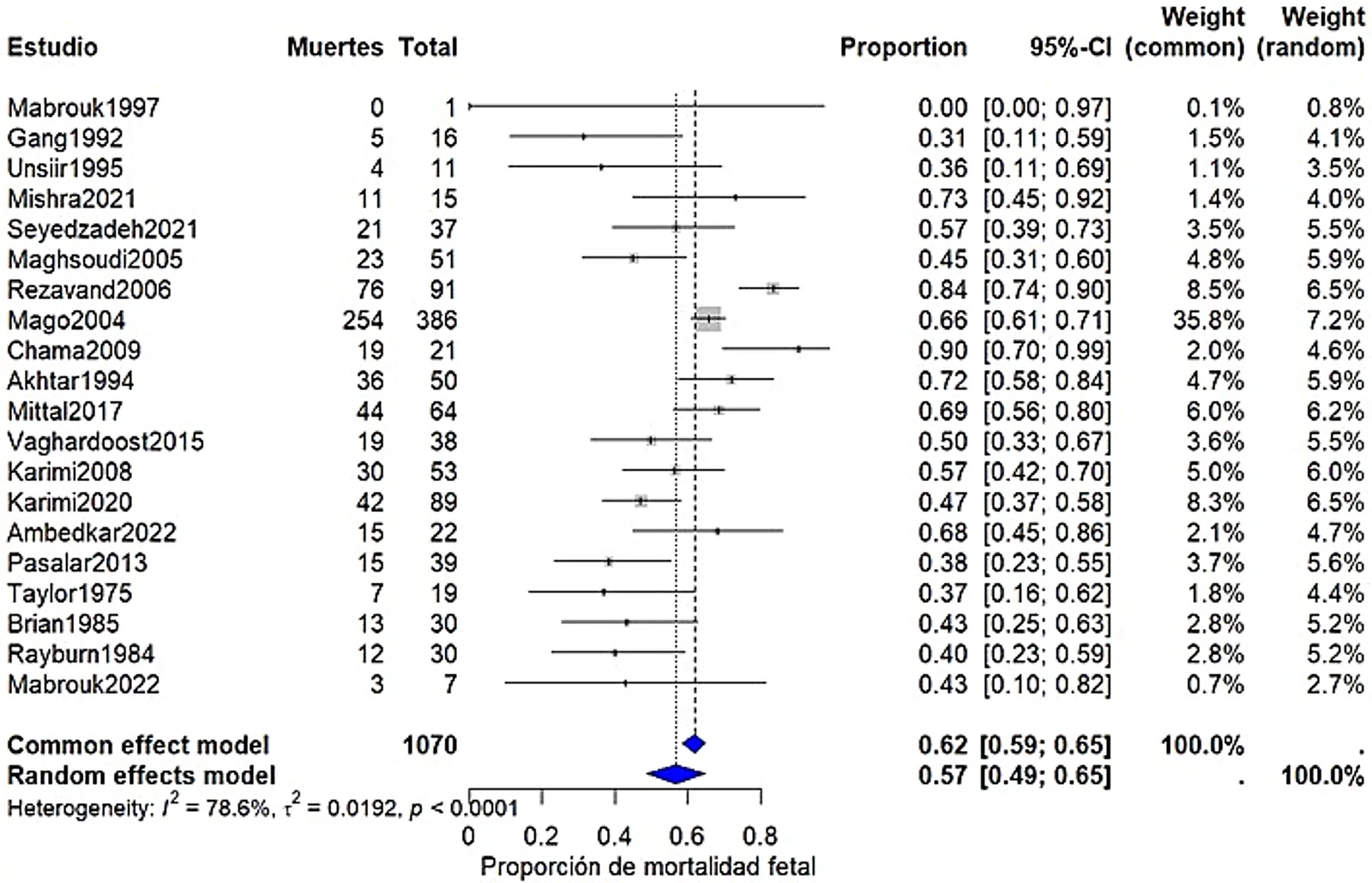
Pooled Proportion of Fetal Mortality in Pregnancies Complicated by Burn Injuries. Forest plot displaying individual study estimates and pooled proportion of fetal mortality in pregnancies complicated by burn injuries. A random-effects model (DerSimonian–Laird) with Freeman–Tukey double arcsine transformation was applied. Horizontal lines indicate 95% confidence intervals, squares represent study-specific weights, and the diamond indicates the pooled estimate.

Using a random-effects model (DerSimonian–Laird) with Freeman–Tukey double arcsine transformation, the pooled fetal mortality proportion was 0.57 (95% CI, 0.49-0.65). The fixed-effect model yielded a slightly higher estimate of 0.62 (95% CI, 0.59-0.65), although the presence of significant heterogeneity supports the use of the random-effects model for inference.

Between-study heterogeneity was considerable (*I*^2^ = 78.6%, *P* < .0001), reflecting substantial variability across included studies. This heterogeneity is likely driven by differences in burn severity (particularly %TBSA burn), gestational age at the time of injury, access to specialized care, and variability in the prevalence of complications such as sepsis and shock.

Studies including a higher proportion of severe burns (TBSA ≥50% burn) consistently reported markedly higher fetal mortality rates.

Overall, the pooled analysis indicates that more than half of pregnancies complicated by burn injuries result in fetal death, underscoring the extreme vulnerability of the fetus in this context and the critical impact of maternal clinical status and burn severity on perinatal outcomes.

Visual inspection of the funnel plots for maternal (Figure 4A) and fetal mortality (Figure 4B) suggests the presence of asymmetry, raising concern for potential publication bias. In both plots, smaller studies (with higher standard errors) appear to be unevenly distributed around the pooled effect estimate, with a relative paucity of studies on one side of the funnel, particularly in regions corresponding to lower mortality estimates.

**Figure 4:**
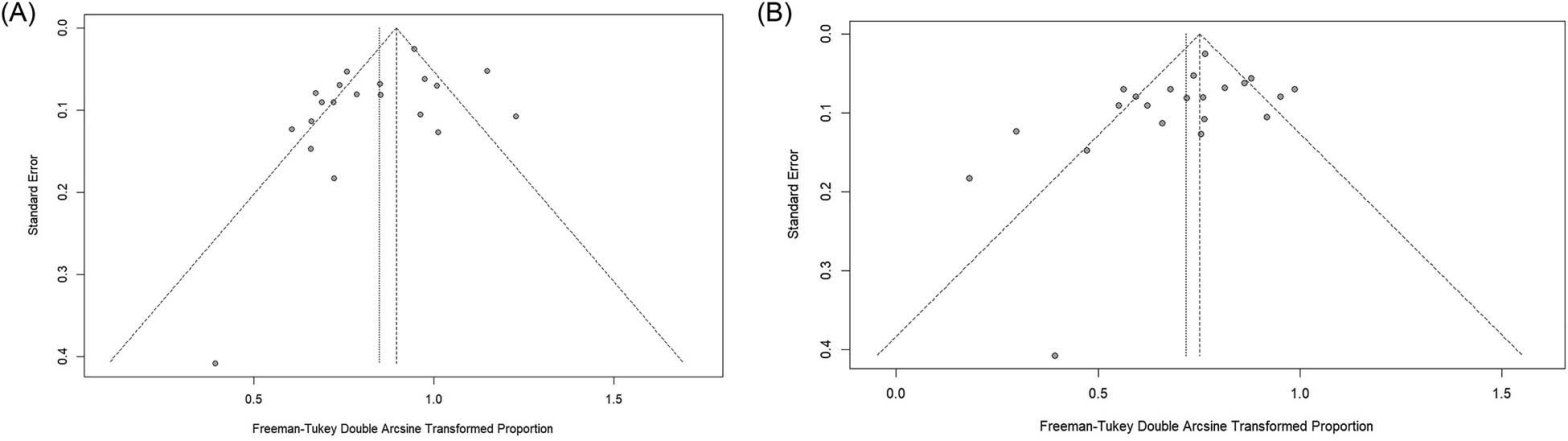
Funnel Plots for Maternal and Fetal Mortality. Funnel plots of studies reporting (A) maternal mortality and (B) fetal mortality in pregnancies complicated by burn injuries. The *x*-axis represents the Freeman–Tukey double arcsine transformed proportion, and the *y*-axis represents the standard error. The vertical line indicates the pooled effect estimate, and the dashed lines represent the 95% confidence limits. Visual inspection suggests asymmetry in both panels, indicating potential small-study effects or publication bias.

This pattern may indicate selective reporting or non-publication of studies with less extreme or null findings. However, the observed asymmetry should be interpreted with caution, as it may also reflect substantial between-study heterogeneity, differences in study size, variability in burn severity (particularly %TBSA burn), and methodological diversity across included studies. Additionally, the predominance of small case series and the limited number of large-scale studies further constrain the interpretability of funnel plot symmetry in this context.

Overall, while the visual patterns are suggestive of possible small-study effects or publication bias, these findings are not definitive and should be considered alongside the high heterogeneity observed in the meta-analyses.

Following the assessment of publication bias, the certainty of the evidence for the main outcomes was evaluated using the GRADE approach. Overall, the certainty of evidence for both maternal and fetal mortality was rated as low. This rating was primarily driven by the observational nature of the included studies and the methodological limitations identified in the risk of bias assessment (Table 2). Most of the included studies were case series, with only a small number of cohort studies, a single cross-sectional study, and one case report, inherently limiting the internal validity of the evidence. Additionally, several studies showed moderate to high risk of bias, mainly due to lack of control for confounding variables, retrospective design, small sample sizes, and incomplete follow-up in some cases.

Further downgrading was applied due to inconsistency. Substantial heterogeneity was observed in both meta-analyses, with I^2^ values of 72.9% for maternal mortality and 78.6% for fetal mortality. This variability likely reflects differences in burn severity, particularly TBSA, as well as variations in clinical management, healthcare resources, and study populations across settings. No serious concerns were identified regarding indirectness, as all included studies directly addressed the target population of pregnant women with burn injuries and reported clinically relevant outcomes. Similarly, imprecision was not considered a major limitation, given that the pooled estimates showed relatively narrow confidence intervals despite the variability across studies.

Publication bias was considered possible based on visual inspection of the funnel plots, which suggested asymmetry for both maternal and fetal mortality outcomes. However, this finding was interpreted with caution due to the limited number of studies and the presence of substantial heterogeneity, both of which can affect the reliability of funnel plot interpretation.

## DISCUSSION

This systematic review and meta-analysis provides a comprehensive quantitative synthesis of maternal and fetal mortality among pregnant women with burn injuries. Across 22 studies including 1162 pregnant women, the pooled maternal mortality proportion was 0.43, whereas the pooled fetal mortality proportion was 0.57. These estimates demonstrate that burns during pregnancy remain a highly lethal condition and confirm that fetal mortality exceeds maternal mortality in most settings. The magnitude of mortality observed in this review is clinically important, particularly because many of the included studies were conducted in low- and middle-income countries, where preventable domestic and intentional burn mechanisms remain common and access to specialized burn, obstetric, and neonatal care may be delayed or limited.^4^^,^^6^^,^^16^^,^^18–20^

The pooled estimates are consistent with the pattern reported across the primary literature. Early studies such as Thermal Injury During Pregnancy and Major Burns During Pregnancy: Effects on Fetal Well-Being first demonstrated that fetal loss, preterm delivery, and maternal death were closely related to burn severity.^6^^,^^7^ Later studies from India, Iran, Nigeria, Egypt, and other settings reinforced these findings, although reported mortality rates varied widely.^3–6^^,^^11^^,^^13^^,^^16^^,^^18–21^ For example, Burns in Pregnancy: Effect on Maternal and Fetal Outcomes reported very high maternal and fetal mortality among patients with extensive burns, while Burns in Pregnancy found that both maternal and fetal mortality increased with larger burn size.^1^^,^^10^ Similarly, Maternal and Foetal Outcome of Burns During Pregnancy in Kermanshah, Iran and Severe Burn Injury in Pregnancy in Northern Nigeria described extremely poor outcomes in women with burns exceeding 50% TBSA.^4^^,^^22^ In contrast, smaller series such as Management of Thermal Injury in Pregnancy: An Analysis of 16 Patients and A Retrospective Study of 11 Pregnant Women with Thermal Injuries reported lower maternal mortality, likely reflecting differences in burn severity, inhalation injury, clinical management, and case mix.^9^^,^^16^

A central finding of this meta-analysis is the consistent association between total body surface area burned and mortality. Although the included studies used heterogeneous %TBSA burn categories, nearly all reported a stepwise increase in maternal and fetal mortality with greater burn extent.^1–5^^,^^8–11^^,^^14^^,^^16^^,^^19^^,^^20^ This relationship supports a dose–response pattern and is biologically plausible. Larger burns are associated with more profound capillary leak, fluid loss, hypovolemic shock, systemic inflammatory activation, infection, and multiorgan dysfunction. In pregnancy, these processes may be amplified by the physiological demands of gestation and may rapidly compromise uteroplacental perfusion.^2^^,^^8^^,^^10^^,^^12^^,^^16^ The findings of Pregnancy and Burns: Guidelines for Safe Management are particularly relevant, as that study identified %TBSA burn as the main determinant of maternal mortality and reported that %TBSA burn together with inhalation injury were major predictors of fetal mortality.^13^ Assessment of Maternal and Fetal Outcomes of Burn in Pregnancy and Feto-maternal Outcomes in Pregnancies Complicated by Thermal Burns also supported %TBSA burn as a key prognostic factor, with progressively worse outcomes in higher %TBSA burn strata.^14^^,^^23^ The higher pooled fetal mortality compared with maternal mortality deserves particular attention. Fetal survival depends almost entirely on maternal oxygenation, perfusion, and metabolic stability. Even when the mother survives, transient episodes of hypotension, hypoxemia, acidosis, sepsis, or severe inflammatory response may be sufficient to cause placental hypoperfusion and fetal demise.^2^^,^^8^^,^^12^^,^^16^ Major Burns During Pregnancy: Effects on Fetal Well-Being described fetal death occurring early after injury and linked fetal compromise to maternal burn extent and systemic deterioration.^6^ Thermal Injury in the Pregnant Patient similarly emphasized that fetal death may occur despite maternal survival, particularly in cases complicated by hypotension, respiratory distress, sepsis, or electrolyte disturbances.^8^ This pattern was also evident in several more recent series, including Foetomaternal Outcome in Pregnancy with Burn Injury: A Prospective Cohort Study, Assessment of Maternal and Fetal Outcomes of Burn in Pregnancy, and Maternal and Fetal Outcome of Burn During Pregnancy: 3rd Report from Kermanshah, Iran, where fetal losses remained frequent even when maternal mortality was lower.^5^^,^^12^^,^^14^

Gestational age appears to modify fetal prognosis and clinical decision-making, although the available evidence was insufficient for robust subgroup meta-analysis. Earlier gestational injuries are more commonly associated with spontaneous abortion or intrauterine fetal death, while later gestational injuries require complex decisions about fetal monitoring, timing of delivery, and neonatal viability.^9^^,^^15^^,^^16^ Obstetric Implications of Burns in Pregnancy, Management of Thermal Injury in Pregnancy: An Analysis of 16 Patients, and A Retrospective Study of 11 Pregnant Women with Thermal Injuries all emphasized that management should be individualized according to burn severity, maternal stability, and fetal maturity.^9^^,^^15^^,^^16^ In viable third-trimester pregnancies, early delivery may be considered when maternal status is stabilized and fetal compromise is suspected.^9^^,^^15^^,^^16^ However, the evidence remains based on small observational series, and no standardized protocol can be inferred from the current data. This highlights the need for multidisciplinary management involving burn surgeons, obstetricians, intensivists, anesthesiologists, neonatologists, and mental health professionals when appropriate.^9^^,^^13^^,^^15^^,^^16^

Inhalation injury and sepsis were additional factors repeatedly associated with adverse outcomes. Inhalation injury may worsen maternal hypoxemia and increase fetal exposure to carbon monoxide, thereby impairing oxygen delivery.^2^^,^^8^^,^^12^^,^^16^ Burn Injuries During Pregnancy in Iran and Burns in Pregnancy both reported strong associations between inhalation injury and maternal or fetal death.^1^^,^^2^ Sepsis was one of the most frequently reported causes of maternal death, particularly in studies from resource-limited settings, including Evaluation of Maternal and Foetal Outcomes in Pregnant Women Hospitalized in Kermanshah Hospitals, Iran, Owing to Burn Injury, 2003-2008, Maternal and Fetal Outcome of Burn During Pregnancy: 3rd Report from Kermanshah, Iran, and Pregnancy and Burns: Guidelines for Safe Management.^12^^,^^13^^,^^24^ These findings reinforce that early resuscitation alone is insufficient; survival also depends on infection prevention, timely wound closure, appropriate antimicrobial stewardship, nutritional support, and intensive care capacity.^8^^,^^12^^,^^13^^,^^16^^,^^24^ The sociocultural dimension of burns during pregnancy is also important. Several included studies reported a considerable proportion of intentional injuries or self-immolation, frequently associated with kerosene, gasoline, domestic conflict, poverty, limited education, and gender-based vulnerability.^2–4^^,^^25^^,^^26^ Burnt Pregnant Wives: A Social Stigma explicitly framed burns among pregnant women as a social problem, while Burn Injuries During Pregnancy in Iran, Epidemiology of Burns During Pregnancy in Tehran, Iran, and Epidemiology of Burns During Pregnancy in Southern Iran: Effect on Maternal and Fetal Outcomes found that suicidal or self-inflicted burns were associated with larger %TBSA burn and worse maternal-fetal outcomes.^2^^,^^25^^,^^26^ Foetomaternal Outcome in Pregnancy with Burn Injury: A Prospective Cohort Study also identified domestic violence and suicide attempts as important contributors.^5^ Therefore, prevention cannot rely solely on clinical interventions; it must also address household safety, access to safe cooking fuels, mental health, domestic violence, education, and social support for pregnant women.^2–5^^,^^25^^,^^26^

The substantial heterogeneity observed in this meta-analysis is expected and clinically meaningful. Heterogeneity likely reflects differences in %TBSA burn distribution, burn depth, inhalation injury, trimester at injury, mechanism of burn, intentionality, time to presentation, and availability of specialized burn and neonatal care.^1–21^^,^^23^ Differences in study design and reporting also contributed. Most included studies were retrospective case series, and many did not provide standardized definitions of fetal death, neonatal survival, sepsis, inhalation injury, or burn severity categories.^1^^,^^2^^,^^5–8^^,^^12^^,^^15^^,^^17^^,^^18^^,^^21^^,^^23^ This heterogeneity supports the use of random-effects models and cautions against interpreting the pooled estimates as a single universal risk applicable to all settings. Instead, the estimates should be interpreted as a summary of a highly variable but consistently severe clinical problem.

The funnel plots suggested possible publication bias or small-study effects for both maternal and fetal mortality. This finding should be interpreted cautiously because funnel plot asymmetry in meta-analyses of proportions may be caused by true clinical heterogeneity rather than selective publication. Nevertheless, the possibility that severe cases and high-mortality series are more likely to be reported cannot be excluded. Conversely, mild burn injuries during pregnancy may be underrepresented because they may be managed as outpatients or not published, which could inflate pooled mortality estimates. This limitation is important when applying the findings to all pregnant women with burns, including those with minor injuries.^7^^,^^8^^,^^12^^,^^15^^,^^23^

The overall certainty of evidence was low. This was mainly due to the observational nature of the evidence, the predominance of case series, moderate to high risk of bias in several studies, inconsistent reporting, and high heterogeneity.^1–21^^,^^23^ However, the direction of association between %TBSA burn and mortality was remarkably consistent across studies, time periods, and geographic regions.^1–5^^,^^8–11^^,^^14^^,^^16^^,^^19^^,^^20^ Thus, although the exact pooled mortality estimates should be interpreted with caution, the conclusion that burn severity is the dominant prognostic factor is strongly supported by the available evidence.

This review has several clinical implications. First, all pregnant women with burns should be evaluated promptly for burn severity, inhalation injury, hemodynamic instability, and fetal viability when gestational age permits.^7–9^^,^^15^^,^^16^ Second, early transfer to specialized burn centers should be prioritized for moderate or severe burns, especially when %TBSA burn approaches or exceeds 20%-30%, when inhalation injury is suspected, or when there is maternal instability.^7–9^^,^^11^^,^^16^ Third, fetal management should be guided by maternal stabilization, gestational age, and fetal monitoring rather than by obstetric intervention alone.^7^^,^^8^^,^^15^^,^^16^ Fourth, because sepsis remains a major cause of death, early wound care, infection surveillance, nutritional support, and timely surgical management are essential.^9^^,^^11^^,^^14^^,^^16^^,^^17^ Finally, prevention strategies must be integrated into maternal health programs, particularly in regions where domestic flame exposure, unsafe fuels, and intentional burns are common.^13^^,^^16^^,^^18–20^ This study also has research implications. Future studies should use standardized reporting frameworks for burns in pregnancy, including maternal age, gestational age, trimester, %TBSA burn, burn depth, mechanism, intentionality, inhalation injury, resuscitation strategy, surgical timing, sepsis, obstetric management, fetal monitoring, and neonatal outcomes.^7–9^^,^^15^^,^^16^ Prospective multicenter registries would be particularly valuable because individual centers rarely treat enough cases to generate precise estimates.^7–9^^,^^15^^,^^23^ Standardized outcome definitions would allow more reliable subgroup analyses by %TBSA burn, trimester, inhalation injury, and management strategy. Given the low certainty of current evidence, collaborative research is essential to develop evidence-informed clinical pathways.

This meta-analysis has important strengths. It provides a focused synthesis of a rare but high-impact condition and includes studies across several decades and multiple geographic settings.^1–21^^,^^23^ It also quantifies both maternal and fetal mortality and integrates risk of bias and certainty of evidence assessment. However, the findings should be interpreted in light of the limitations of the underlying evidence. Most included studies were single-center retrospective series with small samples, incomplete adjustment for confounding, and variable outcome definitions.^1^^,^^2^^,^^5–8^^,^^12^^,^^15^^,^^17^^,^^18^^,^^21^^,^^23^ In addition, the inability to access some full texts and the possibility of underreporting of mild cases may have influenced the pooled estimates. Despite these limitations, the consistency of the relationship between the %TBSA burn and mortality across the literature provides a clinically coherent and actionable message.^1–5^^,^^8–11^^,^^14^^,^^16^^,^^19^^,^^20^ The included studies span approximately 5 decades, during which substantial advances have occurred in burn resuscitation, critical care, obstetric management, fetal monitoring, neonatal intensive care, and infection control. Consequently, the pooled estimates should be interpreted cautiously as maternal and fetal outcomes reported in earlier studies may not fully reflect contemporary clinical practice.

In conclusion, burn injuries during pregnancy are associated with exceptionally high maternal and fetal mortality, with fetal outcomes being particularly poor.^1–21^^,^^23^ The extent of burn injury, measured by %TBSA burn, is the most consistent predictor of adverse outcomes, while inhalation injury, sepsis, delayed care, and intentional injury further worsen prognosis.^3^^,^^4^^,^^9–11^^,^^14^^,^^16^^,^^17^^,^^19^^,^^20^ These findings support early aggressive maternal resuscitation, multidisciplinary care, specialized burn referral, and public health strategies aimed at preventing domestic and intentional burns among pregnant women.^7–9^^,^^11^^,^^13^^,^^16^^,^^18–20^ Although the certainty of evidence remains low, the clinical signal is clear: pregnant women with moderate to severe burns represent an obstetric and burn-care emergency requiring immediate coordinated management.^7–9^^,^^15^^,^^16^

## LIMITATIONS

This study has several important limitations that should be considered when interpreting the findings. First, the overall quality of the available evidence was limited, as the majority of included studies were case series with inherent methodological constraints. The predominance of observational and descriptive designs increases susceptibility to selection bias, information bias, and confounding, thereby limiting the internal validity of the pooled estimates. Second, there was substantial heterogeneity across studies, as reflected by high I^2^ values in both maternal and fetal mortality analyses. This heterogeneity likely arises from differences in burn severity, particularly TBSA, variations in clinical management, healthcare resource availability, and sociocultural factors such as the proportion of intentional injuries. Although a random-effects model was applied, residual heterogeneity cannot be fully accounted for. Third, important clinical variables were inconsistently reported across studies, including gestational age at the time of injury, presence of inhalation injury, timing of intervention, and standardized definitions of outcomes. This limited the ability to perform subgroup analyses and to explore potential sources of heterogeneity in a more robust manner. Fourth, the potential for publication bias cannot be excluded. Funnel plot asymmetry suggested the presence of small-study effects; however, this finding should be interpreted cautiously given the limited number of studies and the predominance of small sample sizes. Forth, most included studies originated from low and middle income countries, where burn severity at presentation, access to specialized burn centers, obstetric care and neonatal intensive care differ substantially from high income settings. Therefore, the pooled estimates may not be fully generalizable to higher resourced countries, where advances in multidisciplinary burn management and perinatal care may improve maternal and fetal outcomes.

## CONCLUSIONS

Burn injuries during pregnancy are associated with high maternal and fetal mortality, with fetal death occurring more frequently than maternal death. Greater burn size, particularly ≥50% TBSA burned, was the most consistent factor associated with poor outcomes across studies. However, the certainty of evidence was low because most data came from observational studies and case series with substantial heterogeneity. These findings support early multidisciplinary management, prompt referral to specialized burn care, and the need for prospective studies with standardized reporting. Further research is needed to better characterize maternal and fetal outcomes, identify modifiable prognostic factors and evaluate the impact of advances in burn, obstetric, and neonatal care. Continued investigation through prospective multicenter studies and standardized reporting frameworks is essential to strengthen the evidence base and guide evidence informed management of pregnant women with burn injuries.

## Supplementary Material

Supplement_1_irag155

## Notes

Author contributions: Juan Jose Parra Molina (Conceptualization [lead], Data curation [supporting], Formal analysis [supporting], Funding acquisition [equal], Investigation [equal], Methodology [equal], Project administration [equal], Resources [equal], Software [equal], Supervision [supporting], Validation [supporting], Visualization [supporting], Writing—original draft [equal], Writing—review & editing [equal]), Edgar Fabian Manrique Hernandez (Conceptualization [supporting], Data curation [lead], Formal analysis [lead], Funding acquisition [equal], Investigation [equal], Methodology [equal], Project administration [equal], Resources [equal], Software [equal], Supervision [supporting], Validation [supporting], Visualization [supporting], Writing—original draft [equal], Writing—review & editing [equal]), and Sergio Romero (Conceptualization [supporting], Data curation [supporting], Formal analysis [supporting], Funding acquisition [equal], Investigation [equal], Methodology [equal], Project administration [equal], Resources [equal], Software [equal], Supervision [lead], Validation [lead], Visualization [lead], Writing—original draft [supporting], Writing - review & editing [supporting])

Funding: The authors report no financial or personal relationships that could have influenced the work reported in this study. This research did not receive any specific grant from public, commercial, or not-for-profit funding agencies.
