# Supplementary material for: Maternal and Fetal Outcomes in Pregnant Women with Burn Injuries: A Systematic Review and Meta-Analysis
Source: J Burn Care Res. 2026 Aug 28;47(6):1746–56. doi: 10.1093/jbcr/irag155 (PMC13732906; doi:10.1093/jbcr/irag155)
Supplement: Supplementary file 1 [file supplement_1_irag155.pdf]

Supplementary Material 1. Detailed search strategies for all databases

Information sources	Search strategy
PubMed/MEDLINE	("Pregnancy"[Mesh] OR Gestation*[tiab] OR pregnan*[tiab]) AND ("Burns"[Mesh] OR "Burns, Chemical"[tiab] OR "Burns, Electric "[tiab] OR "Electric Burns"[tiab]) AND ("Maternal Mortality"[Mesh] OR "Mortality, Maternal"[tiab] OR "Pregnancy-Related Mortality"[tiab] OR "Fetal Mortality"[tiab] OR "Late Fetal Mortality"[tiab] OR "Mortality, Fetal"[tiab])
Scopus	(TITLE-ABS-KEY(pregnan* OR gestation*)) AND (TITLE-ABS-KEY(burns OR "chemical burns" OR "electric burns")) AND (TITLE-ABS-KEY("maternal mortality" OR "pregnancy-related mortality" OR "fetal mortality" OR "late fetal mortality"))
Web of Science	TS=(pregnan* OR gestation*) AND TS=(burns OR "chemical burns" OR "electric burns") AND TS=("maternal mortality" OR "pregnancy-related mortality" OR "fetal mortality" OR "late fetal mortality")
LILACS	(tw:(pregnan* OR gestation*)) AND (tw:(burns OR "quemaduras" OR "chemical burns" OR "electric burns")) AND (tw:("maternal mortality" OR "mortalidad materna" OR "pregnancy-related mortality" OR "fetal mortality" OR "mortalidad fetal"))
